# Impacts of being scolded at workplace on psychological distress among Japanese employees: a cross-sectional study

**DOI:** 10.1539/eohp.2025-0009

**Published:** 2026-03-24

**Authors:** Kohei Mase, Kanami Tsuno, Reiko Kuroda, Kotaro Imamura, Norito Kawakami, Daisuke Nishi, Natsu Sasaki

**Affiliations:** 1 Department of Mental Health, Graduate School of Medicine, The University of Tokyo, Tokyo, Japan; 2 School of Health Innovation, Kanagawa University of Human Services, Kanagawa, Japan; 3 Division for Environment, Health, and Safety, The University of Tokyo, Tokyo, Japan; 4 Department of Digital Mental Health, Graduate School of Medicine, The University of Tokyo, Tokyo, Japan

**Keywords:** constructive feedback, harassment, occupational stress, subordinate, supervisor

## Abstract

**Background:**

While scolding is an often problematic form of workplace communication in Japan, little research exists regarding its impact on employees’ mental health.

**Objectives:**

This study investigated the associations between being scolded at work and psychological distress.

**Methods:**

Data from the Employee Cohort Study during the COVID-19 pandemic in Japan (E-COCO-J) surveys obtained in September 2023 (14th wave) were used. Psychological distress was measured using the Kessler Psychological Distress Scale (K6), with a cutoff of 13 points. Multivariable logistic regression was conducted by adjusting demographic factors, psychiatric treatment history, and workplace factors.

**Results:**

A total of 758 participants were included in the analysis. Among the participants, 159 individuals (21%) reported having been scolded at least one time in the past month, and 116 individuals (15.3%) had a K6 score of 13 or higher. Compared to those who were not scolded, being scolded less than 1 day a week (adjusted odds ratio [AOR] 1.83; 95% confidence interval [CI], 1.07–3.11) and about more than 1 day a week (AOR 2.42; 95% CI, 1.23–4.76) showed significant associations with high psychological distress.

**Conclusion:**

This study suggested that being scolded has a negative impact on employees’ mental health. Further study is needed to investigate effective methods of delivering constructive negative feedback rather than resorting to scolding.

## Introduction

Mental health in the workplace is an important global issue. Approximately 15% of workers worldwide experience mental health challenges, contributing to an estimated annual economic loss of one trillion dollars^1)^. In Japan, workplace mental health concerns are also on the rise. Nearly 60% of workers in Japan report experiencing high levels of stress, and the number of cases recognized as work-related incidents due to psychological burdens from work are on the rise^2)^. Given these growing issues, it is essential to identify and examine workplace factors that contribute to mental health issues.

Communication in the workplace is one of the important occupational factors which are associated with workers’ mental health^1,3)^. For example, supportive communication by supervisors and coworkers, such as respect and dignity, has been found to have a positive impact on mental health^3)^. In contrast, negative acts or communication, such as workplace bullying and incivility, have been found to have detrimental effects on mental health^4,5)^. Negative feedback is one form of negative communication, which is defined as pointing out bad points regarding undesirable or below-expected performance^6)^. It has been reported that employees are more likely to improve their job performance based on negative feedback if it is delivered by a trustworthy supervisor, with high-quality content, and in an effective manner^7)^. However, negative feedback was likely to be rated low in satisfaction^7)^. Frequent negative feedback reduced subordinate performance^8)^ and did not demonstrate a beneficial effect on work engagement, unlike positive feedback^6)^.

In Japanese education research, scolding is considered a form of negative feedback and is defined as “actions where an individual points out to the other party actions that they perceive as unsatisfactory and asks them to improve”^9)^. This study adopts this definition as the core definition of scolding, focusing on the intent of the scolder. In work-related settings, scolding is often generally understood as a subset of negative feedback characterized using verbal expressions aimed at causing a change in the behavior or perception of another person by inducing negative emotional experiences, such as fear, anxiety, pain, or sadness. Anger expression refers to the manner in which individuals typically experience, express, and control anger^10)^ and is sometimes involved with acts of scolding. Yet, the core element of scolding is the explicit intention to promote improvement, rather than merely inducing negative emotions. Scolding is thus different from workplace bullying, which is defined as repeated and systematic negative acts toward an individual over time, leading to a power imbalance between the parties involved^11)^ (full definition available in eMaterial 1). Scolding and workplace bullying differ in that scolding is intended to promote improvement, whereas bullying involves harmful acts and does not necessarily include feedback. Low supervisor social support and scolding are also distinct concepts, since low supervisor support refers simply to the absence of support, whereas scolding includes an active intention to change the counterpart’s behavior, even with negative communication. Thus, scolding a distinct form of workplace communication. The present study, therefore, positions scolding as a concept differentiated from these adjacent concepts.

Scolding is an often problematic form of workplace communication, especially in Japan^12)^. In this context, scolding is more widely accepted as a form of negative feedback in workplace settings. This may be partly attributable to the prevalence of strong hierarchical relationships between superiors and subordinates^13)^. For instance, in Japan, a tendency was observed that people in higher positions expressed anger more frequently than those in lower positions^14)^. However, the impact of workplace scolding is still unknown. Specifically, the extent to which negative feedback, particularly its subset of scolding, impacts psychological distress in the workplace has not been thoroughly investigated. Considering that scolding is a common form of negative feedback communication in Japan, it may lead to reduced self-esteem and mental health issues. However, no studies on this topic have been conducted. Accordingly, it is essential to investigate whether workplace scolding has any health impacts.

This study aimed to examine the associations between scolding exposure and psychological distress among Japanese workers.

## Methods

### Study design and participants

This was a cross-sectional study using data from an online cohort of full-time workers: The Employee Cohort Study in the COVID-19 pandemic in Japan (E-COCO-J) conducted in September 2023. E-COCO-J included full-time workers registered with an online survey company, targeting workers aged 20–59 years who are working full-time from throughout Japan. Among the 4,120 respondents who participated in the online survey in February 2019, up to 1,500 were recruited to participate in the baseline survey of E-COCO-J in March 2020. The 1,448 respondents in the baseline survey were repeatedly invited to answer the survey, and a total of 14 surveys were conducted through September 2023. To ask about experiences of being scolded in the past month, the current study targeted those who had been continuously employed for at least 1 month before the survey. Therefore, among those who agreed to respond to the 14th survey conducted in September 2023, those who indicated they were working at the time of both the 13th survey (conducted between April 14 and April 28, 2023) and the 14th survey (conducted between September 21 and September 29, 2023) were included in the analysis. The data were derived from a longitudinal survey, but a cross-sectional analysis was conducted because there is limited prior research on the impact of scolding on psychological distress, and the appropriate time frame from exposure to outcome is unclear, making it difficult to design a longitudinal analysis. This study was approved after ethical review by the Graduate School of Medicine, The University of Tokyo 10856-(8).

### Measurements

#### Psychological distress

The Kessler Psychological Distress Scale (K6) was used to measure psychological distress^15,S1)^. K6 comprises six items that assess how frequently respondents have experienced symptoms of psychological distress during the past 30 days. Participants can select responses on a scale from 0 (never) to 4 (always), with a maximum possible score of 24. Higher scores indicate more severe psychological distress. The dependent variable was dichotomized based on the K6 scale, with scores of 13 or higher indicating high psychological distress^S2)^.

#### Scolded experience

The frequency of being scolded in the past month was measured using one item to ask, “In the past month, how often have you been scolded at work? Please answer on average during the past month.” The response options were 1 = “not scolded”, 2 = “less than 1 day per week (less than a few days per)”, 3 = “about 1 day per week”, 4 = “about two to three days per week”, and 5 = “more than four days per week”. Data on scolding were collected only in the 14th survey, which was conducted between September 21 and September 29, 2023. This question item was independently developed, and its reliability and validity have not been verified. To capture scolding from various sources, such as customers and supervisors, we focused on asking about the frequency of being scolded at work rather than specifying who delivered the scolding. Due to sample size constraints, the frequency variable was recoded into a three-category variable based on the frequency of being scolded, with the options of “about 1 day per week”, “two to three days per week”, and “four or more days per week” combined into one category.

#### Covariates

To obtain information on whether individuals experienced customer harassment or workplace bullying in the past month, the data was collected from the 14th questionnaire using the following items: “Experienced bullying or workplace bullying at the workplace, whether related to the new coronavirus or not” and “Received verbal or physical abuse or unreasonable demands from users or customers at the workplace, whether related to the new coronavirus or not.” The response options were 1 = “Yes” and 2 = “No”. This question item was independently developed, and its reliability and validity, including content validity (ie, whether the item was understood as intended), have not been verified. Data on supervisor support, coworker support, job demand (quantity and quality), and job control were obtained from the 14th survey using the Brief Job Stress Questionnaire (BJSQ)^S3)^.To improve intuitive understanding, the quantitative and qualitative job demand scales were adjusted so that higher scores represent greater job demands. Similarly, the job control items were structured to ensure that higher scores reflect greater autonomy in the workplace.

Gender (men, women), age (20–29, 30–39, 40–49, ≥50 years), educational status (high school or less, vocational school or junior college diploma, bachelor’s degree, master’s degree, others or unknown), marital status (single, married), and occupation (manager, manual, nonmanual) were obtained.

### Statistical analysis

Analysis of covariance was conducted to compare the mean levels of psychological distress across groups defined by the frequency of being scolded. Demographic variables (gender, age, educational status, marital status, and occupation), experiences of customer harassment or workplace bullying, history of psychiatric treatment, supervisor support, coworker support, work quantity demands, work quality demands, and work control were included as covariates for adjustment. A multivariable logistic regression analysis was performed with the frequency of being scolded in the past month as the independent variable and high psychological distress (measured using K6) as the dependent variable, using the same set of covariates. Additionally, to examine the impact of whether participants had experienced scolding, a similar multivariate logistic regression analysis was conducted. In this supplementary analysis, the frequency of being scolded was recoded into a binary variable (experienced vs. not experienced), and the same multivariable logistic regression model as the main analysis was conducted using this binary variable as the independent variable instead of the original frequency measure. Sensitivity analysis was conducted to evaluate the robustness of the findings. Specifically, we introduced an interaction term between scolding and workplace bullying or customer harassment into the multivariable logistic regression model. This was done for both the binary model (scolding: none vs. any) and the interval-scale model (three frequency categories), using the same set of covariates as in the primary analysis. Statistical significance was set at a two-sided P-value of <0.05, with P-values less than 0.05 considered statistically significant. Statistical analyses were conducted using IBM SPSS Statistics 29.0.2.0 (IBM Corp., Armonk, NY, USA).

## Results

A total of 891 respondents consented to the 14^th^ survey. Of these, 767 were employed on both the 13th and 14^th^ surveys and a total of 758 participants were included in the analysis (eFigure 1). Nine cases with missing values on covariates were excluded from the analysis: all cases involved individuals who were unemployed at the ninth survey wave, when the psychiatric treatment history was assessed, resulting in the missing data.

Participant characteristics and the frequency of being scolded at work in the past month are presented in eTable 1. The participants included slightly more men than women, with the fewest number of subjects in their 20s and most in their 50s or older. The educational status of the participants tended to be a bachelor’s degree or higher. Twenty-one percent of respondents reported experiencing scolding at least occasionally during the past month, while 7.2% experienced scolding at a frequency of once or more per week. In the past month, 4.4% of respondents reported experiencing customer harassment or workplace bullying.

The results of the relationship between the frequency of scolding and K6 scores are shown in Table 1. Analysis of covariance revealed a significant difference in K6 scores among groups (F=8.99, p<0.001). Bonferroni-adjusted post-hoc comparisons showed that the difference between the “not scolded” and “more than 1 day a week” groups was statistically significant (p<0.001), while other pairwise comparisons were not.

**Table 1.** Analysis of covariance for psychological distress categorized by the frequency of scolding and Post-hoc comparisons of psychological distress by frequency of being scolded (N=758)

	n	(%)		K6 scores		Group difference	
			95% CI	Estimated Marginal Mean ^a^	SD	F-value	P-value
Not scolded	599	(79.0)	6.54–9.04	7.79	0.64	8.99	<0.001
Less than 1 day a week (less than a few days a month)	105	(13.9)	7.49–10.55	9.02	0.78		
More than 1 day a week	54	(7.1)	9.10–12.60	10.89	0.91		

I	J	Mean difference ( I - J )	SE	95% CI for the mean difference	P-value ^b^
Not scolded	Less than 1 day a week (less than a few days a month)	-1.23	0.58	-2.63 to 0.17	0.105
	More than 1 day a week	-3.10	0.78	-4.97 to -1.22	<0.001*
Less than 1 day a week (less than a few days a month)	More than 2 days a week	-1.87	0.90	-4.03 to 0.30	0.116

CI, confidence interval; K6, Kessler Psychological Distress Scale; SD, standard deviation; SE, standard error.

a Estimated marginal means were adjusted for covariates including sex, age, marital status, education, customer harassment/workplace bullying in past month, occupation, supervisor support, coworker support, history of mental illness, job demand (quantity), job demand (quality), job control.

b Bonferroni-adjusted p-values for pairwise comparisons.

The results for associations of being scolded with high psychological distress are shown in Table 2. For the frequency of being scolded, using “not scolded” as the reference category, the adjusted odds ratios (AORs) were as follows: scolded less than 1 day a week (less than a few days a month) (AOR 1.83; 95% confidence interval [CI], 1.07–3.11), and scolded more than 1 day a week (AOR 2.42; 95% CI, 1.23–4.73). Age was associated with lower odds (AOR 0.96 per year increase; 95% CI, 0.94–0.98), and higher job control was associated with lower odds (AOR 0.62; 95% CI, 0.46–0.84).

**Table 2.** Multivariable logistic regression analysis with high psychological distress (K6 ≥13) as the dependent variable (N=758)

	Crude			Adjusted ^b^		
	OR	95% CI	P-value	AOR	95% CI	P-value
Frequency of being scolded in the past month [ref: Not scolded in past month]	1.00			1.00		
Less than 1 day a week (less than a few days a month)	2.57	1.56–4.26	<0.001*	1.83	1.07–3.13	0.028*
More than 1 day a week	3.72	2.01–6.89	<0.001*	2.42	1.23–4.76	0.011*
Education [ref: High school or less]	1.00			1.00		
Vocational school or junior college diploma	0.95	0.56–1.61	0.849	0.99	0.53–1.86	0.989
Bachelor’s degree	0.84	0.53–1.33	0.458	0.79	0.45–1.37	0.397
Master’s degree	0.74	0.29–1.88	0.526	0.72	0.26–2.03	0.535
Others or unknown	0.52	0.17–1.54	0.238	0.83	0.21–3.27	0.787
Married [ref: Single]	0.75	0.51–1.08	0.119	0.85	0.53–1.35	0.486
Women [ref: Men]	0.74	0.51–1.08	0.116	0.80	0.49–1.33	0.401
Age ^a^	0.97	0.95–0.99	<0.001*	0.96	0.94–0.98	<0.001*
Occupation [ref: Manual]	1.00			1.00		
Managers	0.73	0.38–1.38	0.325	1.36	0.63–2.94	0.433
Nonmanual	0.67	0.45–0.99	0.047*	0.92	0.55–1.52	0.730
Customer harassment / workplace bullying in past month [ref: Not received]	2.43	1.21–4.88	0.013*	1.83	0.80–4.15	0.150
Supervisor support ^a^	0.80	0.53–0.92	0.011*	0.93	0.61–1.41	0.728
Coworker support ^a^	0.57	0.43–0.77	<0.001*	0.74	0.49–1.11	0.147
History of mental illness [ref: Absent]	1.72	1.01–2.93	0.047*	1.31	0.67–2.55	0.428
Job demand (quantity) ^a^	1.57	1.22–2.03	<0.001*	1.42	0.99–2.04	0.056
Job demand (quality) ^a^	1.41	1.07–1.86	0.015*	1.02	0.68–1.52	0.923
Job control ^a^	0.55	0.42–0.70	<0.001*	0.62	0.46–0.84	0.002*

AOR, adjusted odds ratio; CI, confidence interval; K6, Kessler Psychological Distress Scale.

* p<0.05.

a For a continuous variable (age, scales), OR for one score increase was shown.

b Analyses were conducted using complete cases only. Analyses were based on participants employed at both the 13^th^ and 14^th^ waves (N=767). After excluding those with missing covariates (N=9), the final analytic sample consisted of 758 participants.

Even when individuals who experienced scolding at least occasionally during the past month were categorized as “experienced” and others as “not experienced,” those who were experienced exhibited significantly higher psychological distress (AOR 2.01; 95% CI, 1.27–3.19; see eTable 2).

The sensitivity analysis using an interaction term between scolding and experiences of workplace bullying showed results consistent with the main analysis. In the binary model, the interaction term was not statistically significant (AOR 1.07; 95% CI, 0.21–5.52; see eTable 3), and being scolded at least occasionally during the past month remained significantly associated with high psychological distress (AOR 2.00; 95% CI, 1.24–3.24; see eTable 3). In the frequency-based model, compared with participants who were not scolded, the adjusted odds ratios for high psychological distress were: AOR 1.87 (95% CI, 1.07–3.27) for “scolded less than 1 day a week (less than a few days a month)” and AOR 2.29 (95% CI, 1.11–4.74) for “scolded about 1 day a week or more” (see eTable 4). The interaction terms in this model were not statistically significant (p=0.822 and 0.722, respectively; see eTable 4).

## Discussion

This study demonstrated that workplace scolding was associated with high psychological distress among Japanese employees, even after adjusting for confounding factors. The findings suggested that scolding may negatively impact workers’ mental health. Notably, even after accounting for workplace bullying —a related but distinct concept — scolding remained a significant factor. Sensitivity analyses indicated that the association between scolding and psychological distress remained consistent regardless of whether individuals had experienced bullying or customer harassment. These findings suggest that scolding may have a unique and independent impact on workers’ mental health. This preliminary evidence underscores the importance of workplace communication in employees’ mental health. Even with corrective intent, scolding may heighten distress, and supervisors should recognize this.

Higher frequencies of workplace scolding were associated with higher K6 scores. These findings aligned with previous studies showing an increase in mental health problems with greater exposure to workplace bullying^4)^.

### Limitations

This study has several limitations. First, this was a cross-sectional study, and causal relationships were unclear. It is possible that reverse causality may be at play, with decreased mental health leading to lower productivity and resulting in being scolded. In addition, employees with high distress may, due to their negative cognition, have perceived routine feedback as scolding. Second, generalizability was limited because it is a panel survey. There is a possibility of bias among the participants, making it difficult to generalize to Japanese labor. Third, due to the dropout of participants from the original cohort, selection bias may have occurred. In addition, the age of the participants was biased toward older workers because the survey was conducted continuously for nearly 4 years. Fourth, we did not provide the definition of “being scolded” to the participants in this study, so it is unclear which specific types of negative feedback were perceived as scolding. Therefore, the scolding measured in this study may have included expressions of anger. This overlap should be taken into account when interpreting the findings. Additionally, the distinction between scolding and workplace bullying was not explicitly presented in the questionnaire. As a result, some participants may have interpreted scolding as bullying, leading to potential overlap in classification. Also, we did not ask respondents who the perpetrators were, but the effects of being scolded may be different depending on who scolded. Further research is needed to clarify the conceptual boundaries between scolding, bullying, and other forms of negative acts in the workplace. Fifth, there is a possibility of unmeasured confounding variables. For example, personality traits, which can affect exposures and outcomes, were not available in this study. We calculated the E-values for the adjusted odds ratios: the E-value for the association between being scolded less than 1 day per week and high psychological distress was 3.06, and for being scolded more than 1 day per week was 4.25. These values suggest that an unmeasured confounder would need to be associated with both the exposure and outcome by a risk ratio of at least this magnitude, above and beyond the measured covariates, to explain the observed associations. Sixth, some of the covariates, such as occupational characteristics and history of treatment for mental illness, were taken from earlier survey waves (2020 and wave 9, respectively). Therefore, there may be potential changes in participants’ status between the time of measurement and the time of the current analysis. Seventh, the questionnaire did not assess the number of times they were scolded within a single day. As a result, the frequency categories may underestimate their impact on psychological distress. Finally, this study did not conduct a priori power calculations. A post hoc estimation was performed using a simplified two-group comparison (high vs. low frequency of scolding), based on the survey results (incidence, 15.3%; α=0.05; power=0.80) due to the complexity of estimating power for multivariate models with multiple exposure categories and covariates. The required sample size to detect an odds ratio of 2.0 was 412 participants (206 per group), and 150 participants (75 per group) for an odds ratio of 3.0. Since the high-frequency group in our study included only 54 participants, the statistical power in that group may have been insufficient.

Despite the many limitations, the fact that individuals who were scolded at least occasionally in the past month showed a significant association with high psychological distress suggests that even a single instance of being scolded per month may have an impact on mental health.

## Supplementary Material

Supplementary eFigure 1 and eTables 1-4 and eMaterial 1-2

## Data Availability

the data supporting this study’s findings are available from the corresponding author, NS, upon reasonable request.

## Approval of the research protocol

This study was approved by the Research Ethics Committee of the Graduate School of Medicine/Faculty of Medicine, The University of Tokyo, No. 10856-(8).
