# Supplementary material for: Impacts of being scolded at workplace on psychological distress among Japanese employees: a cross-sectional study
Source: Environ Occup Health Pract. 2026 Mar 24;8(1):2025-0009. doi: 10.1539/eohp.2025-0009 (PMC13293774; doi:10.1539/eohp.2025-0009)
Supplement: Supplementary file 1 — Supplementary eFigure 1 and eTables 1-4 and eMaterial 1-2 [file eohp-8-2025-0009-s001.pdf]

eFigure 1. Flowchart of participant recruitment

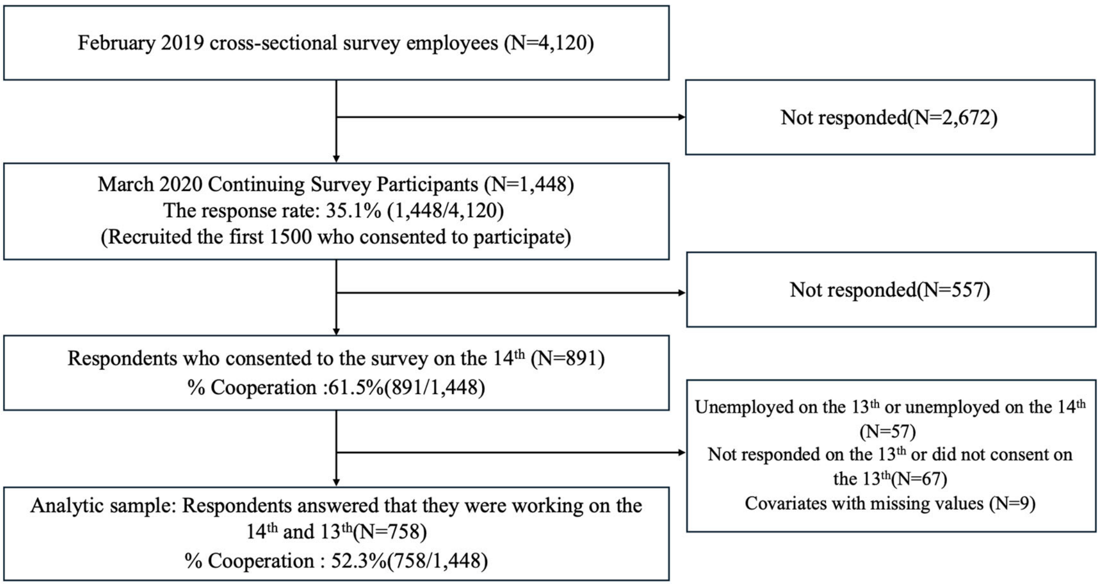

eTable 1. Participant characteristics (N=758)

	Total		not scolded		scolded	
	N (%)	Mean (SD)	N (%)	Mean (SD)	N (%)	Mean (SD)
Gender			599 (100)		159 (100)	
Men	419 (55.3)		320 (53.4)		99 (62.3)	
Women	339 (44.7)		279 (46.6)		60 (37.7)	
Age		47.0 (10.0)		47.6 (9.99)		44.9 (9.57)
20–29 years old	22 (2.9)	28.0 (1.1)	17 (2.8)	27.9 (1.03)	5 (3.1)	28.0 (1.41)
30–39 years old	170 (22.4)	34.1 (2.7)	124 (20.7)	34.1 (2.68)	46 (28.9)	34.2 (2.65)
40–49 years old	214 (28.2)	44.3 (2.8)	166 (27.7)	44.4 (2.88)	48 (30.2)	44.1 (2.55)
≥50 years old	352 (46.4)	56.0 (4.1)	292 (48.7)	56.2 (4.09)	60 (37.7)	55.1 (3.95)
Education [a]						
High school or less	182 (24.0)		138 (23.0)		44 (27.7)	
Vocational school or junior college diploma	169 (22.3)		143 (23.9)		26 (16.4)	
Bachelor’s degree	344 (45.4)		269 (44.9)		75 (47.2)	
Master's degree	42 (5.5)		33 (5.5)		9 (5.7)	
Others or unknown	21 (2.8)		16 (2.7)		5 (3.1)	
Marital status						
Married	417 (55.0)		328 (45.2)		89 (44.0)	
Single	341 (45.0)		271 (54.8)		70 (56.0)	
Occupational type [b]						
Nonmanual	437 (57.7)		356 (59.4)		81 (50.9)	
Manual	229 (30.2)		172 (28.7)		57 (35.8)	
Managers	92 (12.1)		71 (11.9)		21 (13.2)	
History of mental illness [b]						
Present	682 (90.0)		537 (89.6)		145 (91.4)	
Absent	76 (10.0)		62 (8))		14 (8.8)	
Customer harassment/workplace bullying in past month						
Not received	725 (95.6)		582 (97.2)		143 (89.9)	
Received	33		17		16	

	(4.4)		(2.8)		(10.1)	
Frequency of being scolded at work in the past month						
Not scolded	599 (79.0)		599 (100)		0 (0)	
Less than 1 day a week (less than a few days a month)	105 (13.9)		0 (0)		105 (66.0)	
About 1 day a week	31 (4.1)		0 (0)		31 (19.5)	
2 to 3 days a week	18 (3.1)		0 (0)		18 (11.3)	
More than 4 days a week	5 (0.7)		0 (0)		5 (3.1)	
Job demand (quantity) [1 - 4]		2.59 (0.77)		2.51 (0.74)		2.54 (0.69)
Job demand (quality) [1 - 4]		2.60 (0.70)		2.91 (0.79)		2.81 (0.68)
Job control [1 - 4]		2.57 (0.74)		2.62 (0.74)		2.36 (0.73)
Supervisor support [1 - 4]		2.14 (0.71)		2.15 (0.72)		2.09 (0.66)
Coworker support [1 - 4]		2.26 (0.69)		2.28 (0.69)		2.16 (0.71)
K6		5.54 (5.89)		4.86 (5.53)		8.11 (6.49)
Score＜13	642 (84.7)	3.60 (3.82)	528 (88.1)	3.36 (3.79)	114 (71.1)	4.73 (3.76)
Score ≥13	116 (15.3)	16.29 (3.07)	71 (11.9)	16.1 (2.97)	45 (28.3)	16.7 (3.22)

K6, Kessler Psychological Distress Scale; SD, standard deviation.

[a] The variable was measured from the 2nd survey (conducted between May 22 and May 26, 2020) and the 3rd survey (conducted between August 7 and August 12, 2020)

[b] The variable was measured from the 9th survey (conducted between February 21 and February 28, 2022)

eTable 2. Auxiliary dichotomized analysis with high psychological distress (K6 ≥13) as the dependent variable (N=758)

	Crude			Adjusted [b]		
	OR	95% CI	P-value	AOR	95% CI	P-value
Frequency of being scolded in the past month [ref: Not scolded in past month]	1.00			1.00		
Scolded in past month	3.11	2.08–4.66	<0.001*	2.01	1.27–3.19	0.003*
Education [ref: High school or less]	1.00			1.00		
Vocational school or junior college diploma	0.95	0.56–1.61	0.849	1.00	0.54–1.87	0.995
Bachelor’s degree	0.84	0.53–1.33	0.458	0.79	0.46–1.39	0.418
Master's degree	0.74	0.29–1.88	0.526	0.71	0.25–2.01	0.523
Others or unknown	0.52	0.17–1.54	0.238	0.86	0.22–3.36	0.833
Married [ref: Single]	0.75	0.51–1.08	0.119	0.85	0.53–1.35	0.484
Women [ref: Men]	0.74	0.51–1.08	0.116	0.81	0.49–1.33	0.397
Age [a]	0.97	0.95–0.99	<0.001*	0.96	0.94–0.98	< 0.001*
Occupation [ref: Manual]	1.00			1.00		
Managers	0.73	0.38–1.38	0.325	1.36	0.63–2.93	0.435
Nonmanual	0.67	0.45–0.99	0.047*	0.92	0.56–1.53	0.751
Customer harassment/workplace bullying in past month [ref: Not received]	2.43	1.21–4.88	0.013*	1.83	0.82–4.19	0.139
Supervisor support [a]	0.80	0.53–0.92	0.011*	0.92	0.61–1.41	0.713
Coworker support [a]	0.57	0.43–0.77	<0.001*	0.74	0.49–1.11	0.138
History of mental illness [ref: Absent]	1.72	1.01–2.93	0.047*	1.33	0.68–2.58	0.404
Job demand (quantity) [a]	1.57	1.22–2.03	<0.001*	1.44	0.99–2.06	0.050
Job demand (quality) [a]	1.41	1.07–1.86	0.015*	1.02	0.69–1.53	0.915
Job control [a]	0.55	0.42–0.70	<0.001*	0.62	0.46–0.84	0.002*

AOR, adjusted odds ratio; CI, confidence interval; K6, Kessler Psychological Distress Scale.

*p<0.05.

[a] For a continuous variable (age, scales), OR for one score increase was shown.

[b] Analysis were conducted using complete cases only. Analyses were based on participants employed at both the 13th and 14th waves (N=767). After

excluding those with missing covariates (N = 9), the final analytic sample consisted of 758 participants.

eTable 3. Sensitivity analysis using an interaction term based on auxiliary dichotomized analysis results

	Sensitivity analysis [b, c]		
	AOR	95% CI	P-value
Frequency of being scolded in the past month [ref: Not scolded in past month]	1.00		
Scolded in past month	2.00	1.24–3.24	0.005
Education [ref: High school or less]	1.00		
Vocational school or junior college diploma	1.00	0.54–1.87	0.993
Bachelor’s degree	0.80	0.46 - 1.39	0.421
Master's degree	0.72	0.25–2.02	0.527
Others or unknown	0.86	0.22–3.37	0.833
Married [ref: Single]	0.85	0.53–1.35	0.488
Women [ref: Men]	0.81	0.49–1.33	0.397
Age [a]	0.96	0.94–0.98	<0.001*
Occupation [ref: Manual]	1.00		
Managers	1.36	0.63–2.93	0.439
Nonmanual	0.92	0.55–1.53	0.750
Customer harassment/workplace bullying in past month [ref: Not received]	1.78	0.54–5.86	0.340
Supervisor support [a]	0.92	0.61–1.41	0.710
Coworker support [a]	0.74	0.49–1.11	0.138
History of mental illness [ref: Absent]	1.33	0.68–2.58	0.402
Job demand (quantity) [a]	1.46	0.99–2.06	0.050
Job demand (quality) [a]	1.02	0.69–1.53	0.916
Job control [a]	0.62	0.46–0.84	0.002
Interaction: Being scolded × Customer harassment/workplace bullying	1.07	0.21–5.52	0.932

AOR, adjusted odds ratio; CI, confidence interval.

*p<0.05.

[a] For a continuous variable (age, scales), OR for one score increase was shown.

[b] Analyses were conducted using complete cases only. Analyses were based on participants employed at both the 13th and 14th waves (N=767). After excluding those with missing covariates (N=9), the final analytic sample consisted of 758 participants.

[c] The sensitivity analysis uses an interaction term between scolding and experiences of workplace bullying

eTable 4. Sensitivity analysis using an interaction term based on multivariable logistic regression results

	Sensitivity analysis [b, c]		
	AOR	95% CI	P-value
Frequency of being scolded in the past month [ref: Not scolded in past month]	1.00		
Less than 1 day a week (less than a few days a month)	1.87	1.07–3.28	0.028
More than 1 day a week	2.29	1.11–4.74	0.026
Education [ref: High school or less]	1.00		
Vocational school or junior college diploma	0.98	0.53–1.84	0.952
Bachelor’s degree	0.78	0.45–1.37	0.383
Master's degree	0.71	0.25–2.01	0.518
Others or unknown	0.80	0.20–3.19	0.749
Married [ref: Single]	0.85	0.53–1.35	0.487
Women [ref: Men]	0.80	0.49–1.32	0.388
Age [a]	0.96	0.94–0.98	<0.001*
Occupation [ref: Manual]	1.00		
Managers	1.35	0.62–2.93	0.447
Nonmanual	0.93	0.56–1.55	0.770
Customer harassment / workplace bullying in past month [ref: Not received]	1.79	0.55–5.90	0.336
Supervisor support [a]	0.93	0.61–1.43	0.750
Coworker support [a]	0.73	0.49–1.11	0.139
History of mental illness [ref: Absent]	1.31	0.68–2.56	0.422
Job demand (quantity) [a]	1.42	0.99–2.04	0.057
Job demand (quality) [a]	1.02	0.69–1.53	0.914
Job control [a]	0.62	0.46–0.84	0.002
Interaction: Being scolded×Customer harassment/workplace bullying [ref: none]	1.00		
Being scolded Less than one day a week (less than a few days a onth)×Customer harassment/workplace bullying	0.80	0.11–5.60	0.822

Being scolded More than 1 day a week×Customer harassment/workplace bullying	1.47	0.18–12.18	0.722

AOR, adjusted odds ratio; CI, confidence interval.

*p<0.05.

[a] For a continuous variable (age, scales), OR for one score increase was shown.

[b] Analyses were conducted using complete cases only. Analyses were based on participants employed at both the 13th and 14th waves (N=767). After excluding those with missing covariates (N=9), the final analytic sample consisted of 758 participants.

[c] The sensitivity analysis uses an interaction term between scolding and experiences of workplace bullying

eMaterial 1. Full definitions of workplace bullying

Workplace bullying is defined as harassing, offending, or socially excluding someone or negatively affecting someone's work. In order for the label bullying (or mobbing) to be applied to a particular activity, interaction, or process, the bullying behavior has to occur repeatedly and regularly (eg, weekly) and over a period of time (eg, about 6 months). Bullying is an escalating process in the course of which the person confronted ends up in an inferior position and becomes the target of systematic negative social acts. A conflict cannot be called bullying if the incident is an isolated event or if two parties of approximately equal strength are in conflict.

eMaterial 2. Supplemental references

S1. Kessler RC, Andrews G, Colpe LJ, et al. Short screening scales to monitor population prevalences and trends in non-specific psychological distress. *Psychol Med*. Aug 2002;32(6):959-76. doi:10.1017/s0033291702006074

S2. Kessler RC, Barker PR, Colpe LJ, et al. Screening for serious mental illness in the general population. *Arch Gen Psychiatry*. Feb 2003;60(2):184-9. doi:10.1001/archpsyc.60.2.184

S3. Inoue A, Kawakami N, Shimomitsu T, et al. Development of a short questionnaire to measure an extended set of job demands, job resources, and positive health outcomes: the new brief job stress questionnaire. *Ind Health*. 2014;52(3):175-89. doi:10.2486/indhealth.2013-0185
